# A Metagenomic Investigation of the Duodenal Microbiota Reveals Links with Obesity

**DOI:** 10.1371/journal.pone.0137784

**Published:** 2015-09-10

**Authors:** Emmanouil Angelakis, Fabrice Armougom, Frédéric Carrière, Dipankar Bachar, René Laugier, Jean-Christophe Lagier, Catherine Robert, Caroline Michelle, Bernard Henrissat, Didier Raoult

**Affiliations:** 1 URMITE CNRS-IRD 198 UMR 6236, Aix Marseille Université, Faculté de Médecine, 27 Bd Jean Moulin, 13385, Marseille, France; 2 CNRS, Aix Marseille Université, UMR7282 Enzymology at Interfaces and Physiology of Lipolysis, 13009, Marseille, France; 3 Hepato-gastroenterology Department, Hôpital de la Timone, Marseille, France; 4 Architecture et Fonction des Macromolécules Biologiques, Centre National de la Recherche Scientifique, Aix-Marseille Université, 13288, Marseille, France; 5 Department of Biological Sciences, King Abdulaziz University, Jeddah, Saudi Arabia; Western University of Health Sciences, UNITED STATES

## Abstract

**Background:**

Few studies have tested the small intestine microbiota in humans, where most nutrient digestion and absorption occur. Here, our objective was to examine the duodenal microbiota between obese and normal volunteers using metagenomic techniques.

**Methodology/Principal Findings:**

We tested duodenal samples from five obese and five normal volunteers using 16S rDNA V6 pyrosequencing and Illumina MiSeq deep sequencing. The predominant phyla of the duodenal microbiota were *Firmicutes* and *Actinobacteria*, whereas *Bacteroidetes* were absent. Obese individuals had a significant increase in anaerobic genera (*p* < 0.001) and a higher abundance of genes encoding Acyl-CoA dehydrogenase (*p* = 0.0018) compared to the control group. Obese individuals also had a reduced abundance of genes encoding sucrose phosphorylase (*p* = 0.015) and 1,4-alpha-glucan branching enzyme (*p* = 0.05). Normal weight people had significantly increased FabK (*p* = 0.027), and the glycerophospholipid metabolism pathway revealed the presence of phospholipase A1 only in the control group (*p* = 0.05).

**Conclusions/Significance:**

The duodenal microbiota of obese individuals exhibit alterations in the fatty acid and sucrose breakdown pathways, probably induced by diet imbalance.

## Introduction

Obesity is a major public health concern resulting from a mixture of environmental, genetic, neural and endocrine factors [1]. The distal gastrointestinal tract harbors >10^14^ microorganisms with significant differences in the taxonomy and concentration of the bacteria along the digestive track reflecting major variations in the environmental niche [1]. There are complex links between the digestive microbiota and obesity, and a new area of research has emerged based on the links between intestinal microbiota, weight change, the relief of malnutrition, and the use of antibiotics and probiotics [2]. The highest bacterial concentration, approximately 10^11–12^ microorganisms per gram of content, resides in the colon and is mainly comprised of anaerobes [1]. In contrast, much lower bacterial concentrations, approximately 10^3–4^ microorganisms per mL of content, are present in the upper two-thirds of the small intestine [3]. *Lactobacillus* sp., *Escherichia coli* and *Enterococci* have been found as the predominant species in the duodenum and jejunum [3,4]. Probiotics and antibiotics can alter the intestinal flora, and the role of *Lactobacillus*, *Bifidobacteria* or *Enterococcus* is easier to understand as the duodenojejunal flora contains mostly these species [2,3]. The small intestine is responsible for most nutrient digestion and absorption by humans. Proteins and lipids are almost completely absorbed before entering the large intestine, along with simple sugars, such as glucose, very few disaccharides (lactose and sucrose), and a portion of starch [5]. In the colon, microorganisms ferment undigested starch (including resistant starch), unabsorbed sugars, plant cell wall polysaccharides and mucins into the short-chain fatty acids (SCFAs) butyrate, acetate and propionate, which provide approximately 10% of the calories humans absorb [5,6].

Human microbiota projects are being initiated throughout the world, with the goal of correlating human physiological phenotypes with the structures and functions of their indigenous microbial communities [7,8]. However, the nature of the changes in the intestinal microbiota associated with obesity is a subject of controversy, and major discrepancies between different studies have appeared [1]. The development of experimental models to study the relationship between gut microbiota and obesity has mostly been based on the study of feces [7]. Recently, we showed that fecal analysis may not be the optimal method to examine the link between obesity and gut flora and that more focus should be given to the microbiota of the small intestine because this is where the calories are absorbed. However, to date, few studies have tested the microbiota of the upper intestinal track [9], and to the best of our knowledge, this compartment of microbiota has been explored by metagenomic analysis only once in humans, namely on ileostomy effluent samples collected from individuals who have had an ileostomy for 20 years [10]. We report here the first metagenomic analysis of duodenal samples from obese and normal volunteers to examine the microbial population and functions of upper intestinal microbiota.

## Materials and Methods

### Human Subjects

Duodenal samples from healthy volunteers were collected in the framework of a clinical study (mrtm02.01) on gastrointestinal lipolysis performed with a solid-liquid test meal. Duodenal samples from obese patients (BMI>28) were collected under similar test meal conditions. These studies were not initially designed for studying gut microbiota at the time they were performed, and samples had been kept frozen in sterile conditions at -80°C since 2003.

### Ethics statement

Experiments were performed at the CPCET (Centre de Pharmacologie Clinique et d'Etudes Thérapeutiques, La Timone Hospital, Marseille) after the clinical protocol was accepted by the institutional review board of the local ethics review committee (CCPPRB, Comité Consultatif de protection des Personnes dans la Recherche Biomédicale, Marseille). Written informed consent was obtained from all participating patients.

### Test meal

The mixed solid/liquid meal used for the clinical studies contained 80 g string beans, 90 g beef, 70 g French fries, 10 g butter, 15 mL olive oil, 5 mL sunflower oil and water for a total volume of 700 mL. Before mixing, the string beans, the beef and the French fries were put into a mincer with 2 mm holes.

### Experimental setup for collecting duodenal samples

After an overnight fasting period, the volunteers/patients were intubated with a single-lumen duodenal tube (outside diameter 5 mm) and a separated single-lumen gastric tube (outside diameter, 3 mm) as previously described [11]. The distal end of the duodenal tube was placed at the ligament of Treitz for aspiration of duodenal contents (-10 cm H2O). The test meal containing a non-abasorbable marker (PEG4000) was introduced into the stomach through the gastric tube using a 50 mL syringe over a period of 5 minutes. The duodenal fluid was then collected continuously by aspiration and fractioned every 15 minutes. Duodenal samples (1 mL) were immediately mixed with 1 mL glycerol and 40 μL protease inhibitors and frozen in liquid nitrogen before storage at -80°C. The samples selected for the present study were all collected at an average time of 90 minutes after meal intake.

### 16S rDNA V6 Pyrosequencing

Total DNA was extracted from the samples using a method modified from the Qiagen stool procedure [QIAamp DNA Stool Mini Kit (Qiagen, Courtaboeuf, France)] [12].

Primers were designed to produce an amplicon length (576 bp) that was approximately equivalent to the average length of reads produced by the GS FLX Titanium (Roche Applied Science, Meylan, France) as previously described [12,13]. The primer pairs commonly used for gut microbiota were assessed in silico for sensitivity to sequences from all phyla of bacteria in the complete Ribosomal Database Project (RDP) database. Based on this assessment, the bacterial primers 917F and 1391R were selected. The V6 region of 16S rRNA V6 was pyrosequenced with unidirectional sequencing from the forward primer with one-half of a GS FLX Titanium PicoTiterPlate Kit 70×75 per patient with the GS Titanium Sequencing Kit XLR70 after clonal amplification with the GS FLX Titanium LV emPCR Kit (Lib-L).

### Metagenomic deep sequencing using Illumina MiSeq

Five samples of weight gain individuals and five samples of normal weight individuals were extracted using the protocol 1 and were amplify by illustra GenomiPhi V2 DN Amplification Kit(GE Healthcare Bio-Sciences Corp. Piscataway, NJ 08855–1327, USA) to get enough genomic DNA. The DNAg of samples were then pooled and sequenced on the MiSeq Technology (Illumina, Inc, San Diego CA 92121, USA) with paired end and barcode according to the Nextera XT library kit (Illumina). The genomic DNA was quantified by a Qubit assay with the high sensitivity kit (Life technologies, Carlsbad, CA, USA) and dilution was performed to require 1ng of each sample as input. The « tagmentation » step fragmented the genomic DNA. Then limited cycle PCR amplification completed the tags adapters and introduce dual-index barcodes. After purification on Ampure beads (Lifetechnolgies, Carlsbad, CA, USA), the libraries were then normalized on specific beads according to the Nextera XT protocol (Illumina). Normalized libraries are pooled into a single library for sequencing on the MiSeq. The pooled single strand library was loaded onto the reagent cartridge and then onto the instrument along with the flow cell. Automated cluster generation and paired-end sequencing with dual index reads was performed in a single 39-hour run in a 2x250-bp. A total information of 6.5 G bases was obtained from a 695K/mm2 density with 91.17% (14,763,000 clusters) of the clusters passing quality control (QC) filters. Within this pooled run, the average index representation was determined 3.80%. The average 478, 239 paired end reads were filtered according to read quality.

### 16S rRNA pyrosequencing analysis

The 16S raw data for all samples was processed and compared using QIIME pipeline 1.7.0 [14], which contains a suite of Python scripts for data analyses. The raw reads were demultiplexed with split_libraries.py and were trimmed using a minimum read length of 150 bp and an average quality score of 30. One mismatch was allowed along the primer sequences. The number of homopolymers authorized in a sequence was limited to 6. The high quality reads were classified to their corresponding operational taxonomic unit (OTU) at 97% similarity using the open-reference OTU picking strategy in QIIME pipeline. The representative OTU sequences were taxonomically classified using the RDP classifier [15] algorithm implemented in QIIME and using the most recent Greengene database gg_12_10 (http://greengenes.secondgenome.com/downloads/database/12_10). The NAST multi-aligner implemented in QIIME performed the multiple sequence alignment that is used to build phylogenic trees. Before building the phylogenic tree, the chimera identification sequence was performed by USEARCH [16]. We also applied a QIIME random subsampling normalization to the OTU table of samples for the sample with the fewest read numbers. Moreover, the OTU table of all samples was filtered, discarding all OTUs that did not contain a minimum of three reads. The QIIME results, including tree, mapping and OTU files, were import into Phyloseq, an R package, for manipulation, alpha indices determination and graph visualizations. The 16S pyrosequencing raw data has been submitted to the SRA archive under the accession number SRP059828.

### Metagenomic analysis

The metagenomic paired-reads were assessed for quality (minimum average of 25) and assembled using the Panda package [17]. The prodigal software performed the detection of open reading frames from metagenomic assembled reads (parameter—p = m) data. The KEGG assignment of the ORFs against the genepep KEGG database was provided by BLASTP, with an E-value of 10^−4^, a minimum bit score of 50 and sequence coverage >70. The corresponding tables from geneID to KO numbers, from KO to EC Enzyme or to pathways numbers, as well as the KEGG mapper tool (http://www.genome.jp/kegg/mapper.html) were used to build the different metabolic pathways. The assignment to Cluster of Orthologous Group (COG) was performed using RPS-Blast against the COG position-specific scoring matrix (PSSM) from the NCBI Conserved Domain Database, with a minimum E-value of 10^−4^, a bit score > 50 and sequence coverage > 70. The blast results were parsed for COG numbers that provided a classification into the different COG categories. The metagenomic raw data has been submitted to the SRA archive under the accession number SRP059828.

### Statistical analysis

The relative abundance differences between obese and normal populations were analyzed using the nonparametric (Kruskal-Wallis test) statistical method. For data comparisons, we used EpiInfo version 6.0 software (Centers for Disease Control and Prevention, Atlanta, GA, USA). A *p* value < 0.05 was considered significant. The raw p-values were adjusted using the Benjamini-Hochberg correction.

## Results

### Subject and sample Characteristics

We tested five lean healthy volunteers (3 males) and five obese patients (3 males) (Table 1). Lean subjects had a BMI of 20.7±2.3 kg/m^2^ (19–24.5) and a mean age of 29.6 (26–34), whereas obese subjects had a BMI of 36.8±8.4 kg/m^2^ (28.3–47.2) and a mean age of 39 (26–54). Although the variance of BMI in the obese group was high, the difference in BMI between the two groups was significant (*p*<0.01) and allowed a clear distinction between lean and obese subjects, in the absence of body composition data.

**Table 1:** Patient characteristics.

Subjects	Sampling date	Age	Sex	Body Mass Index
**Lean, healthy volunteers**	11-Mar-03	28	F	19
	02-Apr-03	26	F	24.5
	10-Apr-03	34	M	19
	30-Apr-03	29	M	20.3
	15-May-03	31	M	20.8
**Obese patients**	28-Mar-03	30	M	36
	10-Apr-03	26	F	29.2
	24-Apr-03	37	F	43.5
	07-May-03	54	M	28.3
	28-May-03	48	M	47.2

At the time of collection of the duodenal samples used for the metagenomic study (90 min after meal ingestion), no significant difference (*p*>0.05) in the rate of gastric emptying was observed between lean (68.5 ± 21.9%) and obese (68.4 ± 25.5%) subjects, who received the same test meal. In both cases, around two third of the meal had already been emptied from the stomach and through the duodenum together with gastric, pancreatic and biliary secretions. Changes in microbiota profile due to differences in gastric emptying were therefore unlikely.

### Duodenal gut microbiota

**Fig 1:**
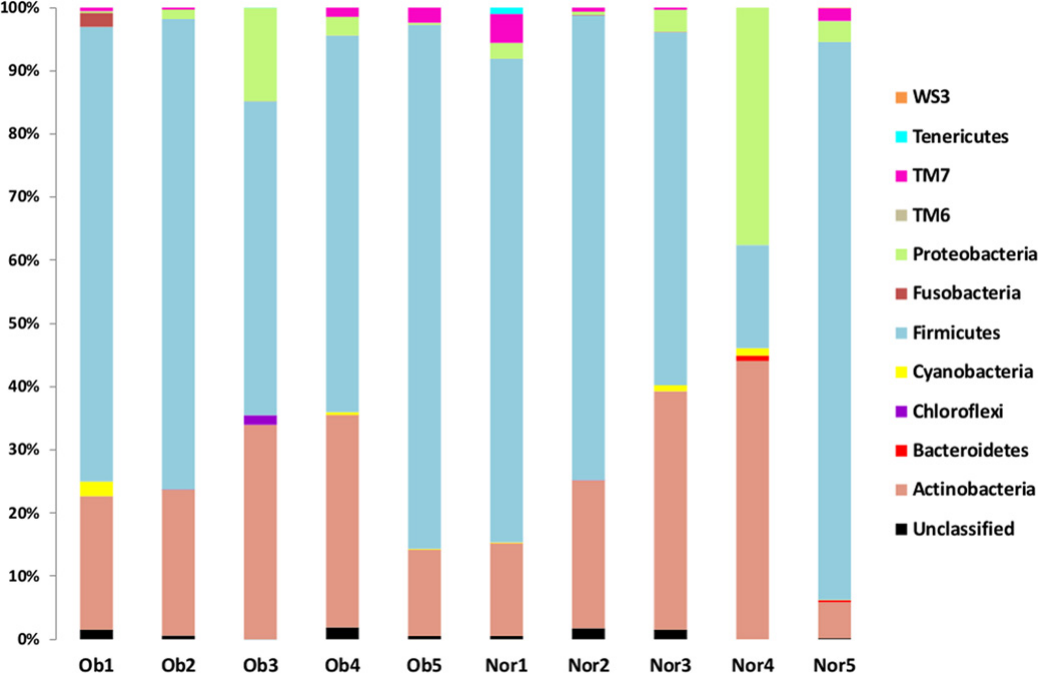
Phylum taxonomic classification. Ob, Obese individual; Norm, Normal weight individual.

**Fig 2:**
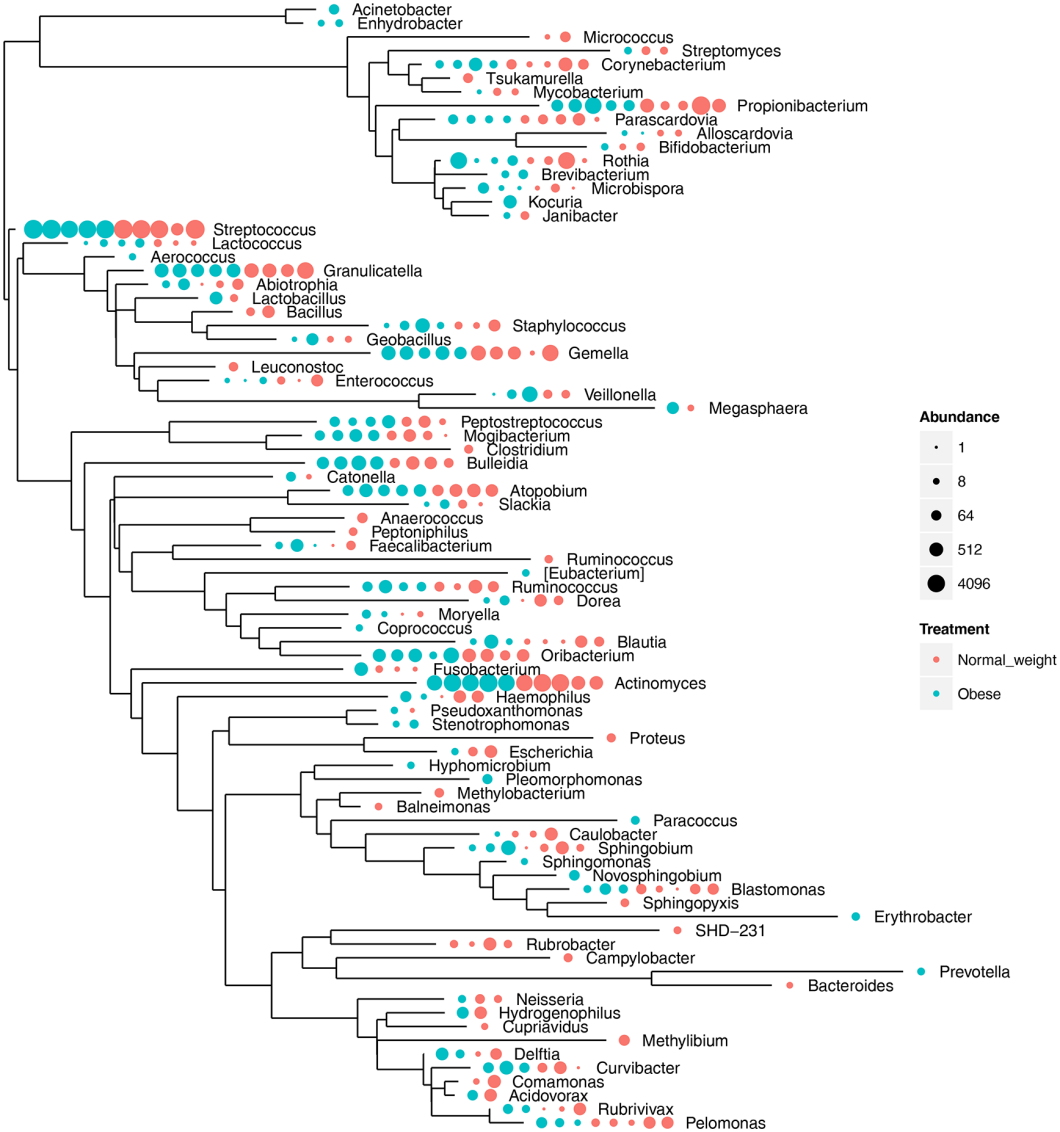
Genus relative abundance by individual. Obese and normal weight individuals are represented by red and blue nodes, respectively. A colored node corresponds to the identification of a genus for one individual. The node size is proportional to the normalized genus abundance.

The microbial structure and composition of the duodenal gut microbiota was characterized by a 16S rRNA pyrosequencing approach. The average 16S rRNA pyrosequencing read length was 374 bp. Table 2 summarizes the number of pyrosequencing-trimmed reads obtained for each of the ten samples. The analysis of the high-quality trimmed reads, which included a random subsampling normalization, indicated that the gut microbiota of the obese and normal individuals was composed of 11 bacterial phyla (Fig 1). Although we observed an inter-individual variability in taxonomic composition, *Firmicutes* and *Actinobacteria* were the most predominant phyla of the bacterial composition of the duodenal microbiota within obese and control groups (Fig 1). These predominant phyla were followed by less abundant ones, including *Proteobacteria*, *Fusobacteria*, TM7, *Bacteroidetes* and *Tenericutes*. Overall, the phylum taxonomic profile was very similar between the obese and control groups (S1 Fig), with small differences for *Firmicutes* (62% in the control group vs 67% in the obese group; *p* = 0.91) and *Proteobacteria* (9.5% in the control group vs 4% in the obese group; *p* = 0.25). Compared with distal gut microbiota, the microbiota of the duodenal site showed major differences exemplified by the almost complete absence of the *Bacteroidetes* phylum (only present at 0.2%) and by the substantial abundance of *Actinomyces* and *Streptococcus* OTUs. We merged OTUs at the genus (Fig 2) and species (S2 Fig) taxonomic ranks to compare the abundance levels for the individuals within each sample category. This comparison between the obese and control categories did not show any significant differences for all tested species and genera except for the *Rubrobacter* genus (*p* = 0.019).

**Table 2:** Standard alpha diversity estimates.

Sample description	No of high quality reads	No. of Observed OTUs (k = 3)	Chao (k = 3)	Shannon (k = 3)
Obese 1	25301	533	596	4.30
Obese 2	35055	528	669	4.27
Obese 3	31769	375	461	4.52
Obese 4	33792	561	628	4.17
Obese 5	25189	559	644	4.67
Normal 1	25762	469	586	4.00
Normal 2	30340	591	655	4.30
Normal 3	25942	556	648	4.30
Normal 4	27069	317	365	4.41
Normal 5	25972	398	496	4.37

Standard alpha diversity estimates were performed from the filtered OTU table.

In this way, the OTUs with less than 3 reads were removed from the initial OTU table.

**Fig 3:**
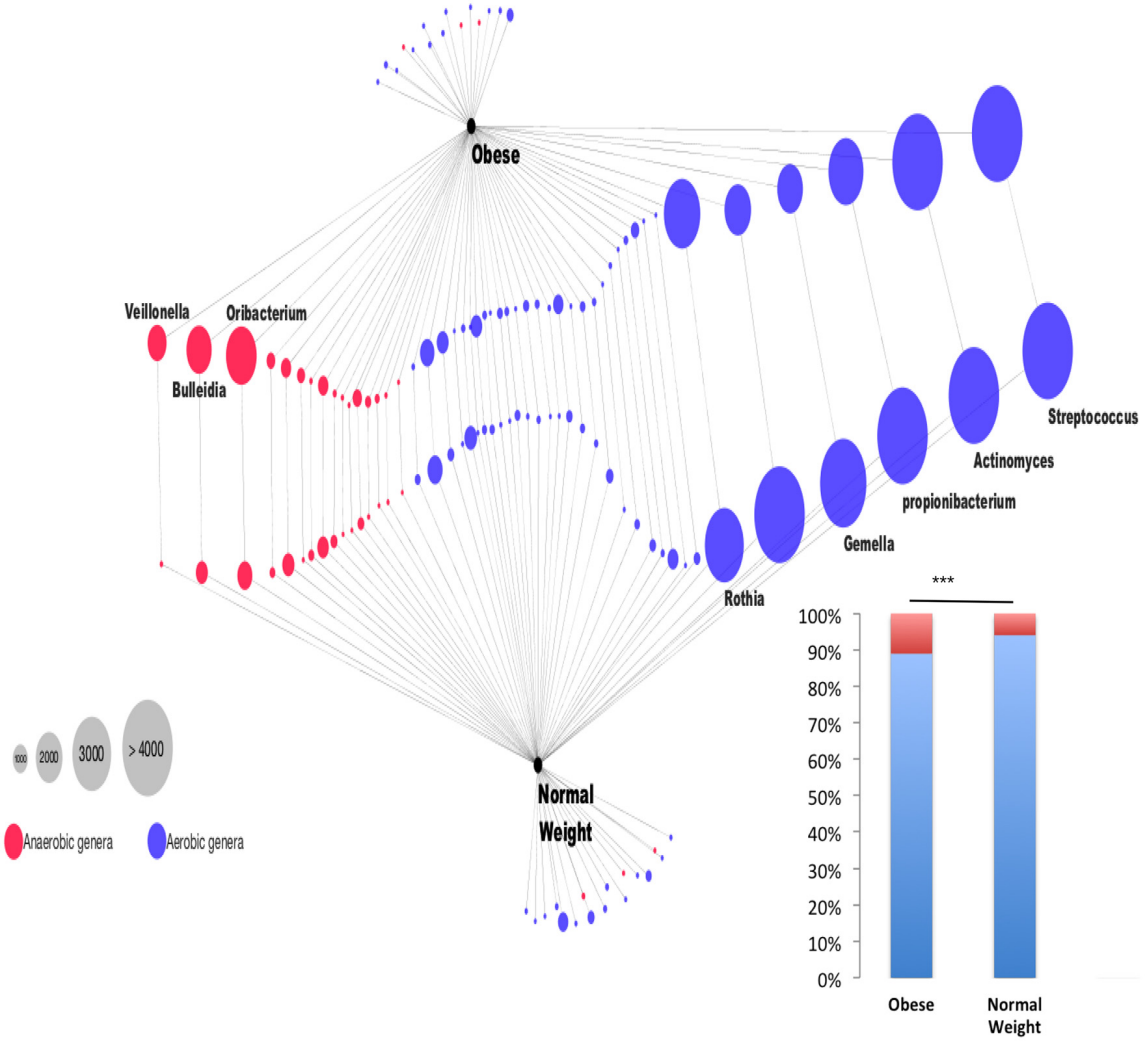
Relative abundance of anaerobic and aerobic genera of the duodenal microbiota. The source black nodes are the obese and the normal weight groups (five individuals by group). The blue and red nodes are the aerobic and anaerobic genera identified in the groups, respectively. The genus node is linked by an edge to it source node. The genus relative abundance is given by the node size. Finally, two genera shared by the obese and normal weight groups are linked by an additional edge. The Cytoscape network visualization tool version 3.1.0 was used for building this figure.

With more observed species, the global richness of the obese group was higher than the control group as shown by rarefaction curves (S3 Fig). However, this difference was not significant using a 3% OTU distance and the Kruskal-Wallis statistical test. In addition, the alpha diversity Simpson indices indicated a weak biodiversity, as only a few abundant OTUs dominated the microbiota composition that mainly comprised *Streptococcus* (30–32%), *Actinomyces* (12–17%), *Propionibacterium* (3–8%) and *Granulicatella* (2–4%) genera. The many remaining OTUs were in weak abundance (most of them < 1%). We also investigated the distribution of aerobic and facultative anaerobic genera residing in the duodenal microbiota of the obese and control groups using the taxonomic classification provided by 16S amplicon analysis. The difference in aerobic genus counts between the obese and control groups was not significant, with 55 and 52 genera identified, respectively. Likewise, the difference in anaerobic genus counts was also not significant, with 55 and 52 genera identified for the obese and control groups, respectively. However, the relative abundance of aerobic and anaerobic genera indicated a significant difference between the obese and control groups (Chi-square test; *p* < 0.001). Compared with the control group, the obese group presented a higher proportion of anaerobic genera and a lesser proportion of aerobic genera (Fig 3).

### Duodenal gut microbiome

Using a metagenomic approach, we investigated the functional capabilities of the duodenal microbiome within obese and control samples. We performed COG (Table 3) and KEGG metabolism category classifications (Table 4). The percentage of COG assigned to carbohydrate metabolism tended to be lower in the obese group than that in the control group (S4 Fig) (*p* = 0.15). In contrast, the proportion of COG assigned to lipid metabolism tended to be higher in the obese group than in the control group (*p* = 0.1). The KEGG analysis gave results equivalent to those of the COG classification, including those for lipid and carbohydrate metabolisms.

**Table 3:** COG assignment by category and individual.

*COG Category (%)*	*VS1*	*VS2*	*VS3*	*VS4*	*VS5*	*OB1*	*OB2*	*OB3*	*OB4*	*OB5*
RNA_processing_and_modification	0	0	0	0.03	0.02	0.13	0.17	0.13	0.02	0.09
Chromatin_structure_and_dynamics	0.02	0.05	0.02	0.03	0.02	0	0.11	0.05	0	0.04
Energy_production_and_conversion	7.59	7.92	9.59	8.03	7.59	8.4	7.75	6.6	7.26	9.95
Cell_cycle_control/cell_division_partionning	1.16	0.98	1.18	1.23	1.07	1.2	0.88	1.09	1.35	1.72
Amino_acid_metabolism_and_transport	9.5	11.28	11.7	10.38	9.65	11.03	9.8	10.3	10.93	9.73
Nucleotide_metabolism_and_transport	3.19	4.13	4.77	4.58	3.42	4.31	3.06	3.74	3.84	3.52
Carbohydrate_metabolism_and_transport	9.71	7.84	9.79	8.42	7.33	7.96	6.73	7.69	7.83	7.5
Coenzyme_metabolism_and_transport	4.23	4.86	5.67	4.84	4.66	4.94	4.25	4.33	5.01	3.17
Lipid_metabolism_and_transport	3.31	2.9	3.46	3.22	2.99	2.8	3.12	3.47	3.64	3.04
Tranlsation_ribosomal_structure_and_biogenesis	9.22	8.83	10.65	9.87	9.74	8.58	8.33	9.34	9.73	10.85
Transcription	5.77	5.46	8.07	5.48	5.45	5.11	5.82	6.12	5.12	5.45
Replication_recombination_and_repair	7.73	8.9	10.45	7.9	8.72	7.69	9.27	9.46	8.57	10.08
Cell_wall/membrane/envelope_biogenesis	5.96	4.48	6.59	5.55	5.73	5.2	6.04	5.79	5.97	4.5
Cell_motility	0.85	0.47	0.54	0.35	0.88	0.71	0.66	0.66	0.52	0.6
Post/translational_modification,_protein_turnover,_chaperone_	4.14	4.32	4.83	4.03	3.56	4.18	4.06	4.38	4.28	3.9
Inorganic_ion_transport_and_metabolism	4.87	4.6	5.75	4.29	5.18	5.16	5.35	4.23	4.47	4.5
Secondary_structure	1.25	1.52	1.68	1.13	1.29	0.84	2.04	1.62	1.4	1.54
General_functional_prediction_only	8.7	9.01	10.47	8.87	9.48	9.6	8.31	8.73	8.59	8.58
Function_unknown	5.63	5.14	6.07	4.93	5.25	5.25	5.49	4.35	4.33	4.85
Signal_transduction	3.38	3.29	4.47	3.68	3.77	2.71	4.25	4.1	3.22	3.39
Intracellular_trafficing_and_secretion_vesicular_transport	1.75	1.77	1.56	1.1	2.05	1.96	1.52	1.39	1.5	0.9
Defense_mechanisms	2.01	2.19	2.64	2.03	2.05	2.13	2.87	2.15	2.33	1.97

**Table 4:** KEGG assignment using BRITE hierarchical classification and by sample group.

	Control group %	Obese group %
**Metabolism**		
Amino_Acid_Metabolism	13.26	13.03
Biosynthesis_of_Other_Secondary_Metabolites	1.69	1.65
**Carbohydrate_Metabolism**	**7.65**	**7.34**
Energy_Metabolism	8.65	8.46
Glycan_Biosynthesis_and_Metabolism	3.64	3.92
**Lipid_Metabolism**	**2.38**	**2.63**
Metabolism_of_Cofactors_and_Vitamins	8.37	8.44
Metabolism_of_Other_Amino_Acids	2.72	2.77
Metabolism_of_Terpenoids_and_Polyketides	1.93	2.34
Nucleotide_Metabolism	4.41	4.12
Xenobiotic_Biodegradation_and_Metabolism	2.21	2.23
**Environmental_Information_Processing**		
Membrane_Transport	8.74	7.81
Signaling_Molecules_and_Interaction	0.02	0.02
Signal_Transduction	2.71	3
**Genetic_Information_Processing**		
Folding,_Sorting_and_Degradation	3.01	3.55
Replication_and_Repair	7.1	7.35
Transcription	2.61	1.95
Translation	9.76	9.24
**Cellular_Processes**		
Cell_Communication	0	0.02
Cell_Growth_and_Death	1.55	1.91
Cell_Motility	0.84	0.81
Transport_and_Catabolism	0.72	0.7
**Human_Diseases**		
Cancers	0.35	0.33
Cardiovascular_Diseases	0.02	0.05
Endocrine_and_Metabolic_Diseases	0.2	0.25
Immune_Diseases	0.07	0.09
Infectious_Diseases	2.45	2.52
Neurodegenerative_Diseases	1.06	0.97
Substance_Dependence	0	0.03
**Organismal_Systems**		
Development	0	0.02
**Digestive_System**	**0.29**	**0.49**
Endocrine_System	0.59	0.74
Environmental_Adaptation	0.41	0.42
Excretory_System	0.01	0.12
Immune_System	0.07	0.11
Nervous_System	0.4	0.35
Sensory_System	0.1	0.2

We investigated the type and the relative abundance of the enzymes involved in lipid pathways, including fatty acid biosynthesis and degradation. In the fatty acid degradation pathway (S5 Fig), we found that the Acyl-CoA dehydrogenase (EC 1.3.99-) targeting the early enzymatic reaction of fatty acid beta-oxidation was enriched (COG and KEGG analyses) in the obese group compared with the control group (*p* = 0.0018). The degradation of a fatty acid requires multiple repetitions of the fatty acid beta-oxidation process (Lynen helix) that leads to the removal each round of two carbon atoms from the acyl chain and to the release of one Acetyl-CoA molecule for the Krebs’ cycle. Moreover, other Acyl-CoA dehydrogenases (EC:1.3.3.6 and EC:1.3.8.3) targeting specific acyl chain lengths but catalyzing the same enzymatic reaction were only detected in the obese group.

The fatty acid biosynthesis pathway (S6 Fig) highlighted the presence of many enoyl-[Acyl carrier protein] reductase enzymes, including FabK, FabI, FabL. These enzymes are components of the type II fatty acid synthase system (Fas) and catalyze the terminal reaction in the fatty acid elongation cycle. The diversity of the enoyl reductase enzymes results from different substrate specificities that can enhance the regulation and the distribution of the products synthetized in the pathway. In our data, FabK was enriched in the control (*p* = 0.027) compared to the obese group; FabL and FabI were only detected in the control group. In addition, the relative abundance of the fatty acid synthase (Fas) tended to be higher in the control group (*p* = 0.07). An inspection of the glycerophospholipid metabolism pathway revealed the presence of phospholipase A1 (EC:3.1.1.32) only in control group (3 of 5 individuals, *p* = 0.05). An analysis of the corresponding protein best BLAST hits indicated that the sequences were homologous to the outer membrane phospholipase A (OMPLA), which is widespread among gram-negative bacteria and is known as a virulence factor in *Campylobacter coli* [18] and *Helicobacter pylori* [19]. In addition, we found that the duodenal microbiota of the obese group showed a reduced abundance of genes encoding sucrose phosphorylase (EC:2.4.1.7) (*p* = 0.015) and 1,4-alpha-glucan branching enzyme (EC:2.4.1.18) (*p* = 0.046), suggesting an alteration of the sucrose/glycogen balance in the obese flora.

## Discussion

We used pyrosequencing of 16S rRNA amplicons and metagenomic analysis to compare the duodenal microbiota in obese individuals to normal weight individuals. Although our samples were collected under similar conditions of test meal and gastric emptying they were taken from a study that was not initially designed for studying gut microbiota, they were kept frozen in sterile conditions at -80°C, eliminating the possibility of contamination. Moreover, before analyses, we verified that all of our samples had a good DNA load. To date, most of the 16S rRNA sequencing- and metagenomic-based studies have analyzed the distal part of the gut using feces and reported differences in the relative abundances of bacterial communities in the gut microbiota of obese versus normal weight people [20]. However, it is noteworthy that the current 16S rDNA studies of gut microbiota within obese populations were not able to detect bacterial concentrations that were below 10^7^ organisms per gram of feces because of sequencing capability limitations [21,22]. As a result, these sequencing-based methods are generally not able to access the complete richness of a feces sample and are biased by the heterogeneity of the copy number of the 16S rRNA gene that is present in an individual bacterial genome, which can lead to an overestimation of bacterial proportions [1,23]. Indeed, the characterization of the 10^11–12^ microorganisms per gram of feces that was used in these studies remains superficial. In contrast with the distal human gut, the bacterial concentrations in the duodenum reach only 10^3–4^ cells per mL of content [3]. Because of the lower bacterial concentration present in the duodenum, we were able to characterize the full species richness of samples by deep sequencing, as shown in the rarefaction curves (S3 Fig). Indeed, when the sequencing effort is adequate to collect the complete species richness of a sample, the curve tends to the asymptote.

A limitation of our study was that we used 12-year-old frozen samples. Excessive degradation of DNA reduces the efficiency of shotgun sequencing [24] and previous studies showed that storing conditions of stool samples modestly affect the structure of their microbial community [25]. It was found that the structure of microbial community is not affected when fecal samples are freeze and stored immediately [25]. Lauber *et al*. reported that the phylogenetic structure of the microbiota did not significantly differ between three and fourteen day old fecal samples stored at a range of temperatures [26]. Moreover it was found that fecal samples kept at -80°C for up to six months also retain a microbiota that was similar in composition to a fresh sample from that individual [27]. Based on these, we believe that the quality and the microbial community of our samples remained stable as all our samples were immediately frozen after collection at −80°C and were never thawed and refreeze.

We found that the duodenal microbiota presents important differences compared to the microbiota of feces. Indeed, the predominant phyla of the duodenal microbiota were *Firmicutes* and *Actinobacteria*, whereas *Bacteroidetes* were almost completely absent. This may be related to a limited availability of mucin as carbon source for *Bacteroidetes*, which are characterized by a high number of genes coding mucin-degrading enzymes like glycosyl hydrolases, proteases/peptidases, sulfatases and sialidases/neuraminidases [28]. Indeed, the mucin layer is much thinner in the small intestine than in the stomach and the colon [28].

Approximately 60% of the genera OTUs belonged to *Streptococcus*, *Actinomyces*, *Propionibacterium* and *Granulicatella*. Similarly, in a recent study, the phylogenetic mapping of the small intestinal metagenome of three different ileostomy effluent samples from a single individual indicated that *Streptococcus* sp., *Escherichia coli*, and *Clostridium* sp. were most abundant in the small intestine [10]. Similarly, in a culture-based study in infants, *Actinobacteria* and *Firmicutes* were found to be the dominant phyla in ileostomy samples, whereas *Bacteroidetes* were only detected following the reversal of the ileostomy in the final fecal sample [29]. In contrast, studies of stool microbiota revealed that *Firmicutes* and *Bacteroidetes* were the predominant phyla [30–32]. Moreover, we found that obese individuals had a significantly higher proportion of anaerobic bacteria in their duodenal microbiota. This difference was mostly associated with the presence of *Veillonella*, *Bulleidia* and *Oribacterium*. Previously, *Veillonella* was detected significantly more in the gut microbiota of children with type 1 diabetes [33]. *Veillonella* are able to ferment glucose and lactate to propionate, acetate and succinate [33].

**Fig 4:**
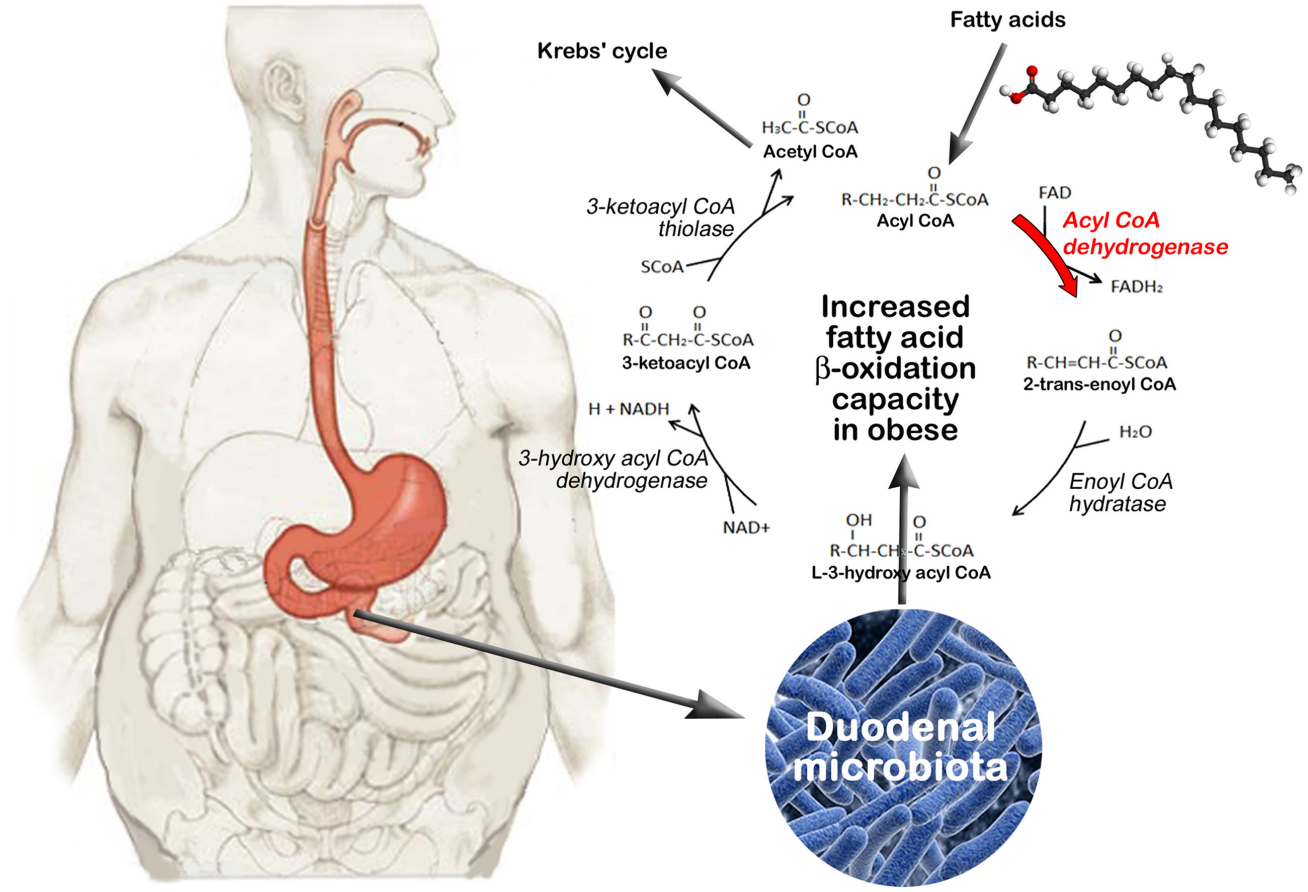
Fatty acid beta-oxidation by duodenal microbiota in obese individuals. The degradation of fatty acids involves their conversion into Acyl-CoA followed by multiple repetitions of the fatty acid beta-oxidation cycle that leads to the removal each round of two carbon atoms from the acyl chain and to the release of one Acetyl-CoA molecule entering in the Krebs’ cycle. Four key enzymes are involved in the beta-oxidation process, including Acyl-CoA dehydrogenase (FAD), enoyl CoA hydratase, 3-hydroxy acyl CoA dehydrogenase and 3-ketoacyl CoA thiolase. FAD, the first enzyme of fatty acid beta-oxidation, was found to be enriched in the microbiota of obese subjects, suggesting a higher beta-oxidation capacity and energy mobilization in these subjects.

The Acyl-CoA dehydrogenase (FAD) involved in the first enzymatic reaction of fatty acid beta-oxidation was enriched in the obese group. This high occurrence of Acyl-CoA dehydrogenase in obese subjects might be associated with a higher beta-oxidation capacity and energy mobilization (Fig 4). Conversely, a higher production of fatty acids would be favored in normal subjects with both a low occurrence of FAD and a high occurrence of OMPLA phospholipase A1. Indeed, OMPLA is particularly active in *E*. *coli* cells with a ‘fad’ mutation and a perturbed cell envelope structure [34]. The *E*. *coli* fad strain does not perform beta-oxidation and is characterized by an appreciable turnover of phospholipids and sizeable amounts of fatty acids resulting from phospholipid hydrolysis [34]. If one assumes that obese patients have an excessive uptake of food and particularly fat, their intestinal microbiota may have adapted to high levels of dietary fats and free fatty acids released upon gastrointestinal lipolysis [35]. Free fatty acids could thus be used as a carbon and energy source for microbial growth. High fat loads are also associated with increased endotoxemia, suggesting that fat and its lipolysis products have a deleterious effect on gut microbiota, leading to LPS release [36–38]. The harvest and degradation of fatty acids by bacteria might be viewed as an adaptive response to their antibacterial effects.

Duodenal sensing mechanisms linked to the release of fatty acids and their levels might also be impacted by microbiota. Fatty acids released in the upper duodenum trigger the release of CCK which first stimulates pancreatic secretion and thus digestion [39]. CCK has however a dual function and also acts as a satiety agent together with other gut hormones like GLP-1 and PYY [40]. It has been suggested that obesogenic microbiota induced high-fat feeding may alters CCK action and lead to dysregulation of glucose homeostasis [41,42]. The mechanism by which obesogenic microbiota may induce CCK resistance has not been explored yet in humans. One hypothesis could be that the microbiota of obese patients may lower fatty acid levels by degrading them more efficiently. This could impact the fatty acid-induced release of gut hormones involved in satiety mechanism and regulation of glucose homeostasis.

In conclusion, the low bacterial concentration and particular taxonomic composition of the duodenojejunal microbiota makes the evaluation of its variation by stool sample analysis extremely difficult. To the best of our knowledge, this is the first time that human duodenal samples have been analyzed by metagenomic techniques, and we found that the duodenal microbiota of obese individuals shows an increased capacity to degrade fatty acids, whereas the flora of control individuals shows an increased capacity to store fatty acids. Because the concentration of living bacteria is much higher in fermented products used as probiotics than in the duodenal flora (10^9^ vs 10^5^ microbes per mL, respectively), the impact of probiotics is probably more important on the duodenal than on the distal gut microbiota.

## Supporting Information

S1 Fig Phylum taxonomic classification by sample group. (DOCX)

S2 Fig Species relative abundance by individual. Each Obese and Normal weight individual are represented by red and blue node, respectively. A colored node is associated to the identification of a species for one individual. The node size is proportionnal to the normalized species abundance.
(DOCX)

S3 Fig Rarefaction curves of observed species. Normal weight and Obese group curves are in red and blue, respectively.
(DOCX)

S4 Fig **A/ Distribution of COG% for Carbohydrate metabolism**. Obese, red color; normal weight, green color. **B/ Distribution of COG % for Lipid metabolism**. Obese, red color; normal weight, green color.
(DOCX)

S5 Fig KEGG Fatty acid degradation pathway. The presence of an enzyme is colored in red or/and green if it is detected in obese or normal weight group, respectively.
(DOCX)

S6 Fig KEGG Fatty acid biosyntethis pathway. The presence of an enzyme is colored in red or/and green if it is detected in obese or normal weight group, respectively.
(DOCX)
