# Supplementary material for: A Metagenomic Investigation of the Duodenal Microbiota Reveals Links with Obesity
Source: PLoS One. 2015 Sep 10;10(9):e0137784. doi: 10.1371/journal.pone.0137784 (PMC4565581; doi:10.1371/journal.pone.0137784)
Supplement: S1 Fig — (DOCX) [file pone.0137784.s001.docx]

**“S1 fig”**
