# Supplementary material for: A Metagenomic Investigation of the Duodenal Microbiota Reveals Links with Obesity
Source: PLoS One. 2015 Sep 10;10(9):e0137784. doi: 10.1371/journal.pone.0137784 (PMC4565581; doi:10.1371/journal.pone.0137784)
Supplement: S2 Fig — Each Obese and Normal weight individual are represented by red and blue node, respectively. A colored node is associated to the identification of a species for one individual. The node size is proportionnal to the normalized species abundance. (DOCX) [file pone.0137784.s002.docx]

**“S2 fig”**
