# Supplementary figures and images for: A Metagenomic Investigation of the Duodenal Microbiota Reveals Links with Obesity
Source: PLoS One. 2015 Sep 10;10(9):e0137784. doi: 10.1371/journal.pone.0137784 (PMC4565581; doi:10.1371/journal.pone.0137784)

**“S3 fig”**


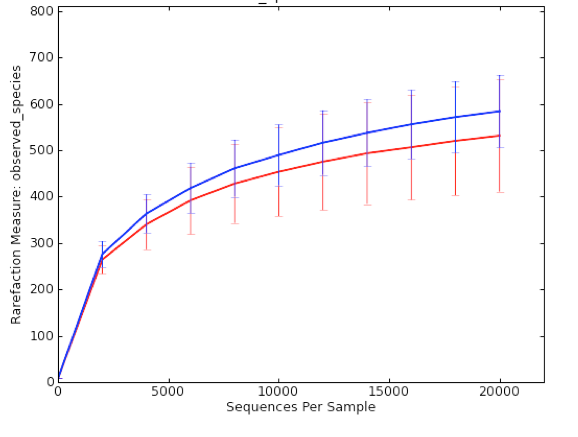

Supplement: S3 Fig — Normal weight and Obese group curves are in red and blue, respectively. (DOCX) [file pone.0137784.s003.docx]

**“S4 fig”**

A


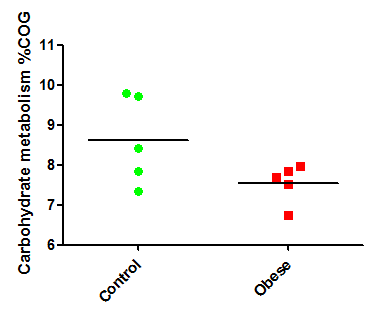


B


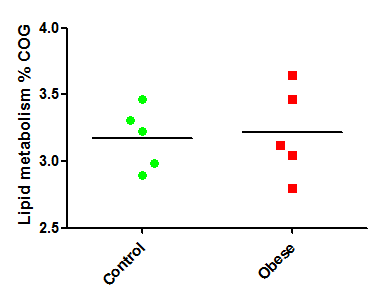

Supplement: S4 Fig — A/ Distribution of COG% for Carbohydrate metabolism. Obese, red color; normal weight, green color. B/ Distribution of COG % for Lipid metabolism. Obese, red color; normal weight, green color. (DOCX) [file pone.0137784.s004.docx]

**“S5 fig”**


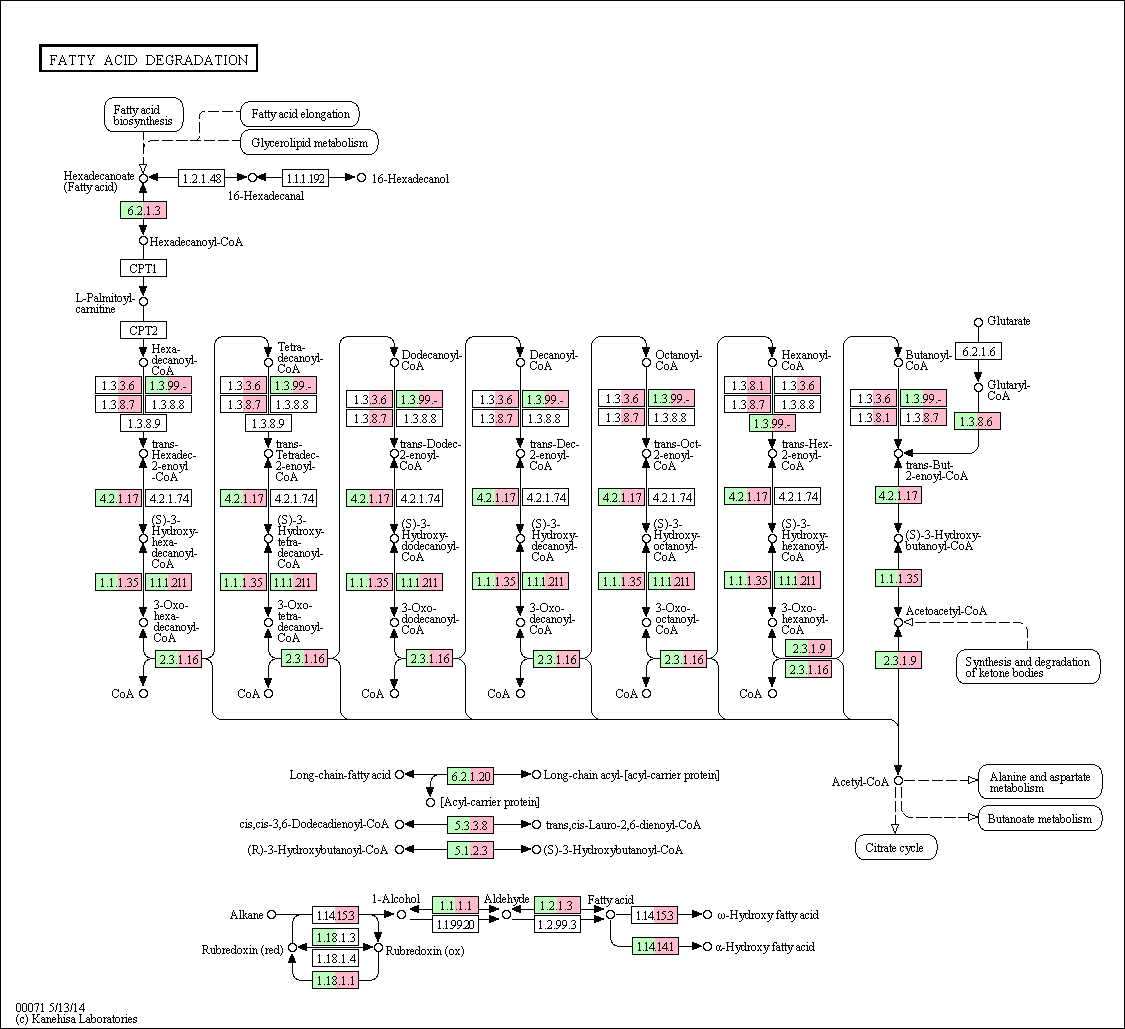

Supplement: S5 Fig — The presence of an enzyme is colored in red or/and green if it is detected in obese or normal weight group, respectively. (DOCX) [file pone.0137784.s005.docx]

**“S6 fig”**


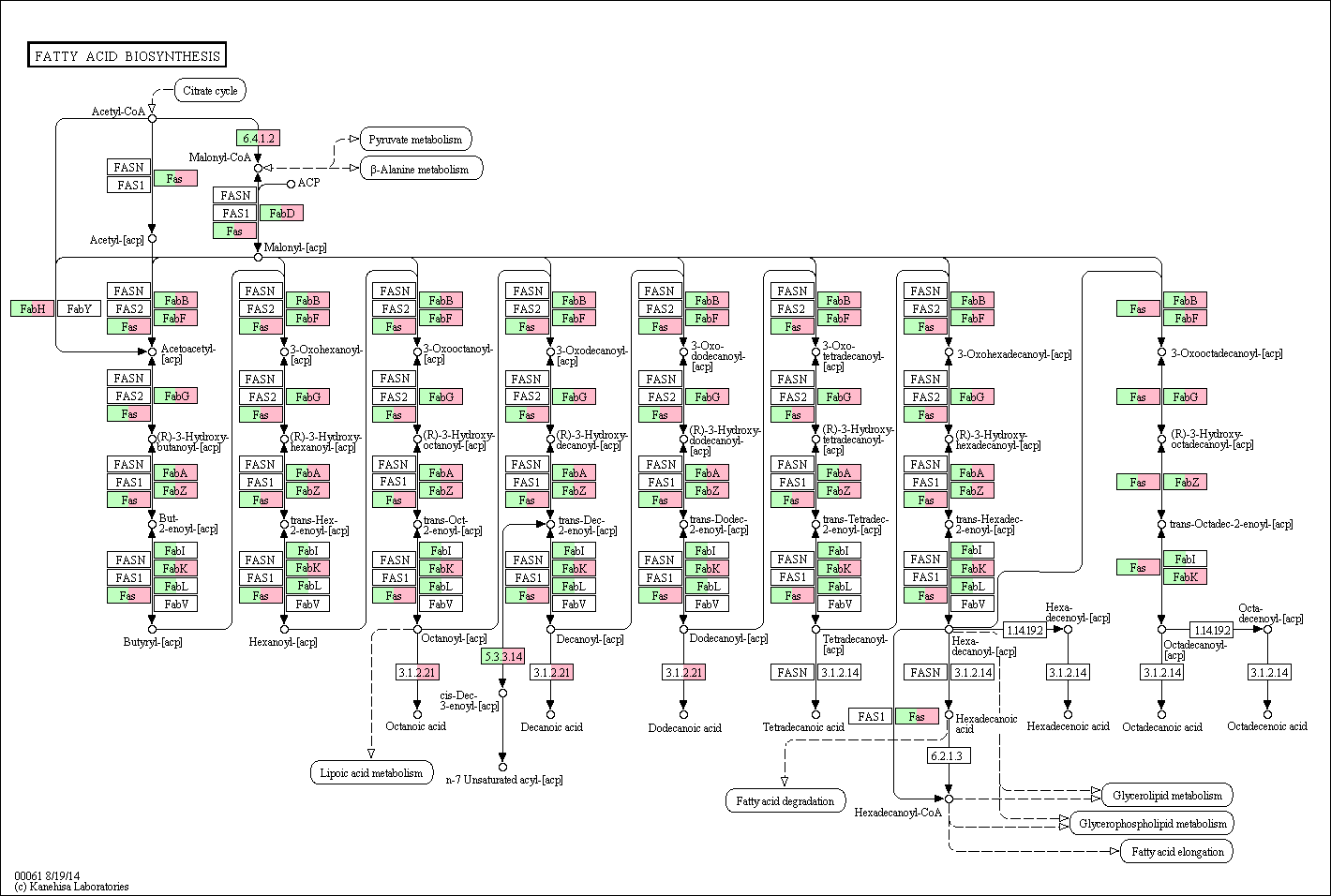

Supplement: S6 Fig — The presence of an enzyme is colored in red or/and green if it is detected in obese or normal weight group, respectively. (DOCX) [file pone.0137784.s006.docx]
